# Emotions evoked by exposure to footstep noise in residential buildings

**DOI:** 10.1371/journal.pone.0202058

**Published:** 2018-08-13

**Authors:** Sang Hee Park, Pyoung Jik Lee, Jeong Ho Jeong

**Affiliations:** 1 Acoustics Research Unit, School of Architecture, University of Liverpool, Liverpool, United Kingdom; 2 Fire Safety & Building Environment System Research Team, Fire Insurers Laboratories of Korea, Yeoju-si, Gyeonggi-do, South Korea; University of Wuerzburg, GERMANY

## Abstract

In the present study, the effect of footstep noise on emotions was investigated. This study used noise stimulus of human footsteps throughout the study. First, Korean emotion lexicons were collected from narratives of residents living in multi-family housing buildings. The lexicons were then classified into four emotion clusters, with three expressing negative emotions (anger, dislike, and pain) and the fourth depicting empathy. Since self-reported annoyance has long been investigated as one of the major non-auditory responses to noise, annoyance was measured along with affective responses in a laboratory experiment with varying noise levels. The findings revealed that the emotion and noise annoyance experienced by the participants were significantly affected by noise levels. All clusters expressing negative emotions showed strong correlations with noise annoyance, whereas that representing empathy showed the weakest correlation. Noise sensitivity and attitudes to the noise source were observed as possible moderators in emotional responses and annoyance ratings.

## Introduction

Emotion is expressed in various forms such as facial expressions and language [1] and it has been commonly investigated by analysing physical and linguistic expressions. Physical analyses of facial expressions and physiological responses [2–6] are useful for identifying emotions of which the perceiver is unaware [7]. However, subtle emotional feelings cannot be determined through physical analyses [8] because of the influences of psychological or physical activities [9]. As another approach, emotion can be studied by examining linguistic expressions. Russell [10] plotted lexicons on a circular model comprising two dimensions (pleasantness and arousal) and showed the interrelationships between the emotions reported. A few studies have also attempted to group emotions on the dimensional model based on their psychological conditions [11–14]. Fehr and Russell [11] conducted series of study to group emotion lexicons under a certain number of prototypes and to validate the grouping procedure. Likewise, Ortony et al. [12] collected a number of lexicons from the literature on emotions and categorised them into eight groups, including physical, affective, and cognitive states. These eight categories were then tested by asking people to rate the emotion lexicons; it was found that the most discriminable categories were affective, cognitive, external, and bodily conditions [13]. Instead of using the dimensional model, Shaver et al. [14] examined the hierarchical structure of emotion concepts and specified prototypes of the emotion categories. They collected 213 emotion lexicons and categorised them into the six groups of love, joy, surprise, anger, sadness, and fear based on subjective ratings [14].

Emotion is a response to a stimulus as well as a quality of excitement that accompanies instinctive reactions [15]. In order to understand the specific emotions provoked by stimuli, different types of stimuli have been used in laboratory experiments. Among them, the most commonly used stimuli are visual images, such as photographs and video clips. For example, Greenwald et al. [16] measured emotions and physiological responses after the presentation of photographic images evoking different emotions (e.g. happy baby or angry face). Acoustic stimuli have also been used to investigate emotions, with variations in sound source and acoustic characteristics. In particular, a majority of acoustic stimuli include outdoor noises such as environmental noise. From a group discussion conducted with participants who were exposed to different noises consisting of environment and neighbour noises, Grimwood [17] reported that three levels of emotional reactions are caused by noises heard at home. Namba et al. [18] developed adjective lexicons in five different languages in order to describe subjective impressions to acoustic stimuli, including road traffic and construction noises. Using cluster analyses, they found that the road traffic and construction noises were grouped together and that the lexicon ‘unpleasing’ was closely tied to ‘annoying’ and ‘noisy’ in most languages [18]. Gomez and Danuser [19] presented 16 environmental noises for 30 seconds in order to evoke broad emotional responses with varying affective valence and arousal. Hume and Ahtamad [20] also used 18 sound clips with a duration of eight seconds each, to investigate pleasantness and arousal, and a majority of the sounds were environmental noises such as traffic and construction noises. In contrast to research on environmental noise, research on emotional responses evoked by indoor noise, such as neighbour noise, is scarce. Although Grimwood [17] investigated emotions related to neighbour noise when studying environmental noise, a majority of the participants were previously exposed to noise from roads, railways, and building works; thus, the emotional reactions to neighbour noise alone were not determined. Stansfeld et al. [21] also pointed out that noise from neighbours is a major source of annoyance and emotional responses in an urban environment; however, its impact has not been studied adequately.

Noise is hazardous to people’s well-being [22]. In particular, exposure to neighbour noise causes annoyance and disturbs activities [23–25]; thus, most studies on neighbour noise have mainly focused on annoyance and sleep disturbance. Raw and Oseland [23] analysed subjective ratings of noise disturbance in conversion flats and reported that noise from upstairs causes sleep disturbance. Through a questionnaire survey and laboratory experiment, Ryu and Jeon [24] explored annoyance caused by indoor noise and the effects of noise sensitivity on annoyance ratings. Park et al. [25] examined the relationship of annoyance caused by floor impact noise with non-acoustic factors such as disturbance and reported that noise annoyance significantly influences health-related complaints. The health risks induced by neighbour noise have been previously reported by several researchers [22, 26, 27]. Additionally, recent laboratory experiments [28, 29] have investigated physiological responses caused by floor impact noise, which is one type of neighbour noise, but emotional reactions were not assessed. Furthermore, neighbour noise results in disputes [30] and even crimes between neighbours [31]. Stokoe and Hepburn [30] analysed discourses of dispute mediation interviews, and the interview extracts clearly showed how residents react to and perceive their neighbours and their noise. Specifically, the interviewees who had disputes with their neighbours described their neighbours as unreasonable, irrational, unaccountable, and distressing [30]. Park [31] reported that the number of registered noise complaints had soared and that there were four murder cases caused by neighbour noise in 2013 in Korea. Park [31] also claimed that such crimes are often retaliatory crimes caused by emotional reactions. Therefore, it is necessary to explore the emotional responses evoked by floor impact noise because annoyance alone may not adequately explain noise-related disputes and crimes.

The above discussion leads us to the following research questions: (1) What kinds of emotional responses other than annoyance are evoked by neighbours’ footstep noise? (2) How are emotional responses related to acoustic and non-acoustic variables such as noise level and noise sensitivity? (3) Can social problems (e.g. neighbour disputes and crimes) be further explained by emotional responses to footstep noise? This study aimed to answer the research questions through online questionnaire surveys and a laboratory experiment. Emotion lexicons on footstep noise were collected from a number of residents’ narratives, which were clustered into different groups by a series of questionnaire surveys. A laboratory experiment was conducted to test the clustered emotional groups with varying sound pressure levels. Participants with different noise sensitivity scores took part in the experiment.

## Emotion classification

### Lexicon collection

Korean emotion lexicons were collected from narratives of residents living in multi-family housing buildings in South Korea. First, interview transcripts from a previous study [32] were used to collect emotion lexicons regarding footstep noise. The interviews were carried out with 14 residents (five males and nine females) living in multi-family housing buildings; their ages ranged from 21 to 55 years and the length of residency in their houses ranged from 10 months to 15 years [32]. Expressions towards their neighbours’ footstep noise, such as ‘bothered’, ‘painful’, and ‘tolerable’, were identified in the transcripts. The second source of lexicons was online communities. As listed in Table 1, posts on a total of 18 different online communities were searched. Nine online communities concerned general topics, such as food, sports, and children, so the members were not restricted to residents of multi-family housing buildings. On the other hand, the other nine communities were limited to residents living in multi-family housing buildings. Lexicons about footstep noise were collected by using the keywords listed in Table 2. The two words ‘noise’ and ‘sound’ were used as the main keywords, and seven sub-keywords, such as ‘floor’ and ‘neighbour’, were introduced. First, online posts containing a combination of one main keyword and at least one sub-keyword were retrieved. Posts on other types of neighbour noise (e.g. piano sounds, voice, chair scraping noise, etc.) were then filtered out. All lexicons were screened based on published research on Korean emotion lexicons [33–36]. After this process, a total of 120 lexicons expressing emotions towards neighbours’ footstep noise were extracted.

**Table 1:** Online communities from which emotion lexicons were collected.

Community topic	No.	Launched date	Number of community members^a^	Number of collected posts
General	1	2004.02.26	3,002,761	754
	2	2003.07.11	2,639,542	1,452
	3	2007.03.03	193,842	893
	4	2009.12.31	162,714	230
	5	2006.03.30	126,532	64
	6	2006.08.26	23,813	197
	7	2012.11.19	12,197	41
	8	2004.02.22	5,425	34
	9	2012.10.02	3,339	12
For residents in multi-family housing buildings	10	2005.10.14	20,371	3,867
	11	2010.06.15	4,430	765
	12	2012.10.25	3,816	691
	13	2014.05.12	2,282	192
	14	2011.07.01	1,758	245
	15	2016.07.11	829	96
	16	2014.06.14	645	68
	17	2011.01.11	511	129
	18	2011.12.28	150	34

^a^Date of the number counting: 28/12/2017

**Table 2:** Keywords used for searching online postings. Korean lexicons used in the study can be found from one of supporting materials (S1 Table).

Category	Keywords
Main keyword	noise sound
Sub-keyword	floor between floors; inter-floor neighbour upstairs foot; footsteps running; jumping walking

### Lexicon sampling and clustering: The survey study

The 120 lexicons collected from the interview transcripts and online communities were used as a primary source in the surveys (Survey I and Survey II). The lexicons were sampled and clustered through the surveys. Both surveys were conducted on an online survey platform (QuestionPro). The study complied with all terms of service for the website. The survey invitation was posted on public online communities, potential respondents were then contacted by email and asked to complete the online questionnaire via an embedded link. The invitations clearly stated the following details of the study: (1) the aim of this study is to explore emotions towards indoor noise, (2) respondents should have normal-hearing and be residents of multi-family housing buildings, and (3) respondents need to use headphones as sounds will be presented in the survey. These instructions were again presented on the first page of each survey, along with a consent form. Only those who provided their consent by clicking “I agree” on the first screen were directed to the questionnaire.

#### Noise stimulus

Footstep noises made by a child and an adult were played during the survey. This noise clip was recorded and used in a previous laboratory experiment [28]. The original recording was 10-second long with dominant sound energies at low-frequencies of below 125 Hz. However, the reproduction of low-frequency components was affected by the hearing device used by the respondents.

#### Lexicon sampling: Survey I

In the online questionnaire, the 120 lexicons were listed randomly. The respondents were asked to listen to the noise carefully and to choose lexicons that represented their emotions towards the stimulus. The noise stimulus was played continuously until the respondents completed the questionnaire. A total of 133 residents (53 males and 80 females) volunteered to take part in Survey I. Sixty lexicons were chosen based on the frequencies, and they were used in the subsequent survey.

#### Lexicon clustering: Survey II

Sixty lexicons chosen from Survey I were randomly presented to the respondents in Survey II. As in Survey I, respondents evaluated the appropriateness of the lexicons for the noise stimulus. The respondents were asked to carefully listen to the noise and to rate the extent to which each lexicon was appropriate for expressing their emotions towards the stimulus, using a 5-point scale (1 = ‘*not at all*’ and 5 = ‘*extremely*’). As listed in Table 3, a total of 89 respondents (43 males and 46 females) took part in Survey II.

**Table 3:** Information about the respondents in Survey II (*n* = 89).

		Number of participants
Age group	20s	4
	30s	10
	40s	49
	50s	21
	60s or over	5
Gender	Male	43
	Female	46
Past experience of neighbour disputes regarding noise	Yes	50
	No	39

In the present study, the cluster analysis method was adopted to classify the lexicons based on the respondents’ ratings. A hierarchical clustering analysis was performed based on the average linkage algorithm and Euclidean distances between lexicons using SPSS for Windows (version 22.0, SPSS Inc. Chicago, IL). Based on the results, 60 lexicons were classified into four clusters (E1, E2, E3, and E4). Emotion lexicons in E1 were mainly related to ‘ANGER’ (e.g. angry, vengeful), those in E2 mostly expressed ‘DISLIKE’ (e.g. unpleasant, bothered), and those in E3 mainly expressed ‘PAIN’ (e.g. painful, distressing). Finally, emotion lexicons expressing ‘EMPATHY’ were grouped in E4 (e.g. bearable, indifferent). As presented in Table 4, E1 had the most lexicons, with 21 lexicons. This may imply that exposure to footstep noise predominantly induces emotions related to anger. As listed in Table 5, the top 20 lexicons were chosen based on the mean scores and they were used in the subsequent laboratory study. There were five, six, four, and five lexicons in E1, E2, E3, and E4, respectively.

**Table 4:** Sixty lexicons grouped in four clusters.

Emotion cluster	Number of lexicons	Emotion prototype	Sample lexicons
E1	21	ANGER	get angry, get enraged, detestable, resent, fury, vengeful
E2	10	DISLIKE	awkward, bothered, irritated, unpleasant, unwelcome
E3	16	PAIN	my head is throbbing, feeling sick, painful, suffering, tired
E4	13	EMPATHY	bearable, just being patient, no reason to get irritated, tolerable

**Table 5:** Twenty lexicons used in the laboratory study. Korean lexicons used in the study can be found from one of supporting materials (S1 Table).

Emotion cluster	Median	Mean	SD	Lexicon
E1	4	3.4	1.3	unhappy
	3	3.2	1.3	detestable
	4	3.2	1.3	can’t understand
	3	3.0	1.4	get enraged
	3	2.9	1.3	ridiculous
E2	4	3.7	1.2	bothered
	4	3.6	1.3	unwelcome
	4	3.5	1.3	dislike
	4	3.4	1.3	get on my nerves
	4	3.4	1.3	awkward
	3	3.3	1.3	vexed
E3	3	3.3	1.4	suffering
	4	3.2	1.3	tired
	4	3.2	1.3	my head is throbbing
	3	3.0	1.4	painful
E4	3	2.9	1.3	bearable
	3	2.9	1.2	just being patient
	3	2.9	1.3	tolerable
	3	2.8	1.4	no reason for discomfort
	2	2.8	1.4	think of it as usual

## Laboratory experiment for the evaluation of emotions

### Methods

#### Noise stimuli

The same noise stimulus (i.e. footstep noise) used in the online surveys was used in the laboratory experiment. As mentioned earlier, it had dominant sound pressure levels at low-frequencies. The noise levels of the stimulus were edited in terms of the A-weighted maximum noise level (*L*_AFmax_), to cover a range from 30 to 60 dB in 5 dB intervals; thus, seven noise stimuli were created. The duration of the stimuli was set to 80 seconds. The 10-second long noise clips were edited to be repeated for 80 seconds.

#### Apparatus

The laboratory experiment was conducted in a sound-proof room with a low background noise level (~25 dBA) in the Fire Insurers Laboratories of Korea (FILK). The floor area was about 35.7 m^2^ (4.8 m × 7.43 m), which simulates the area of a living room in most common apartments. There was a sofa in the middle of the space, a television in front of the sofa, and an air-conditioner on the front wall. The volume of the room was 93.8 m^3^ (4.8 m × 7.43 m × 2.63 m), and the shape of the room was rectangular. Most surfaces were covered with sound absorbers, and the reverberation time of the room was about 0.21 seconds at 1 kHz.

Noise stimuli were reproduced using loudspeakers and a subwoofer in order to replicate real footstep noise. Sounds above 63 Hz were presented via one loudspeaker (Genelec 8050A), while low-frequency sounds below 63 Hz were presented via the subwoofer (Velodyne MicroVee). A low-pass filter with a cut-off frequency of 63 Hz in the octave band was applied to the sounds played via the subwoofer. The loudspeaker and subwoofer were placed in front of the participants, with the loudspeaker mounted at 1.2 m above the floor to simulate the noise from upstairs neighbours. An additional loudspeaker was used to present ambient noise at 31 dBA.

#### Participants

In order to assess statistical power, the sample size in previous research was referred to. Park and Lee [29] previously measured self-rated annoyance when their participants (*n* = 21) were presented with floor impact noise stimuli and found strong correlations between annoyance and noise level (r = 0.95). With this effect size, the sample size was estimated using G*Power to obtain 80% power with α = .05 [37, 38]. The results showed that a total sample of *n* = 34 was needed. Based on this estimation, it was aimed to recruit a minimum of 35 participants and a total of 41 Korean participants (22 males and 19 females) took part in the study. A participant information sheet and a written consent form were provided to the participants upon arrival, and only those who provided their consent participated in the study. None of the participants reported hearing disabilities. Before the experiment, each participant was asked to answer several questions about demographic information, noise sensitivity, and attitude towards their upstairs neighbours. Noise sensitivity was evaluated using 21 questions [39], and attitude towards their upstairs neighbours was assessed using six questions. The questions can be found from one of supporting materials (S1 File). As shown in Table 6, the majority of the participants were in their 30s and 40s. Half of them had one or more children, and more than half reported that they had lived in their current dwelling for less than five years. In order to observe a clear difference between the low and high noise-sensitivity groups, participants with moderate noise sensitivity levels were excluded from the grouping. First, participants’ noise sensitivity scores were divided into five groups using 20th, 40th, 60th, and 80th percentiles from the observed mean score distributions as cut-off points. Second, the middle range between the 40th and 60th percentiles was excluded. Thus, the low and high noise sensitivity groups included individuals with scores lower than the 40th percentile and scores higher than the 60th percentile, respectively. The mean noise sensitivity score of the low group was 79.6 (std. deviation = 6.3), and that of the high group was 102.1 (std. deviation = 6.4). The low and high noise-sensitivity groups had 15 and 16 participants, respectively. Similarly, positive and negative attitude groups were also divided while excluding the middle range between the 40th and 60th percentiles. Those whose attitude scores were lower than 16 were included in the negative attitude group, while those who reported an attitude score higher than 18 were included in the positive attitude group. The mean attitude score of the positive group was 23.4 (std. deviation = 3.6), and that of the negative group was 13.6 (std. deviation = 1.6). The positive and negative attitude groups contained 15 and 16 participants, respectively.

**Table 6:** Information about the participants of the laboratory study (*n* = 41).

		Number of participants
Age group	20s	5
	30s	13
	40s	20
	50s	3
Gender	Male	22
	Female	19
Child(ren) at home	Yes	20
	No	21
Length of residency	less than 1 year	7
	1–3 years	12
	3–5 years	13
	5–10 years	1
	10–15 years	8
Past experience of making noise complaints	No	28
	Yes	13

#### Procedure

A laboratory experiment was conducted to investigate the effect of noise levels on emotions in terms of lexicons. The experiment was designed based on the hypothesis that noise levels would influence emotion and annoyance ratings. Each participant was guided to sit on a sofa in the middle of the room in a comfortable position, and he/she responded to questionnaires on emotion and annoyance ratings while noise stimuli were presented for 80 seconds each. All the stimuli and lexicons listed on the questionnaires were presented randomly to minimise the order effect.

The participants were asked to rate 20 emotion lexicons on 7-point scales (0 = ‘*not at all*’ and 6 = ‘*extremely*’) according to the following instruction: ‘Please rate the extent to which each lexicon is appropriate for expressing your emotions towards the noise you are currently hearing’. Participants were also asked to rate the noise annoyance perceived due to each of the noise stimuli. Participants were provided with the instruction ‘Please rate noise annoyance perceived by the noise you are currently hearing’. Participants used a 7-point scale (0 = *‘not at all’* and 6 = *‘extremely’*) to indicate their level of annoyance. The ratings of emotions and noise annoyance were then translated into a scale from 0 to 100 for assessments of percentage of high emotion rating (%HE) and percentage of highly annoyed (%HA). Both measures were defined as the percentages of emotion and annoyance responses which exceeded a certain cut-off point. Schultz [40] used a cut-off of 72 in his synthesis to define %HA and the same cut-off point was chosen in the present study for both %HE and %HA.

Participants were instructed to consider everything that they heard and felt during noise exposure. Since 10 seconds of footstep noises were repeated over an 80-second period, participants could listen to the stimuli several times when they were responding to the questions. Prior to the commencement of the experiment, each participant attended a trial session to familiarise themselves with the experimental setting in which they responded using 7-point numerical scales.

This study was approved by the School of the Arts Committee on Research Ethics, University of Liverpool. A local ethics committee does not exist; thus, approval was obtained from the local institution where the laboratory experiments were conducted.

#### Data analysis

Statistical analyses were performed using SPSS for Windows (version 22.0, SPSS Inc. Chicago, IL). The main effects of noise levels on the participants’ responses were analysed using the repeated measures analysis of variance (ANOVA), and group differences were examined using the Mann-Whitney U test and independent samples t-test. In the present study, *p* values of less than 5% (*p* < 0.05) were considered as statistically significant. The data used for the statistical analysis can be found from one of supporting materials (S2 File).

### Results

Noise levels caused significant effects on all emotion and annoyance ratings. In addition, the interaction effect between noise levels and noise sensitivity showed significant effects on ratings, while no interaction effect was found between noise levels and attitudes. The effects of noise level, noise sensitivity, and attitudes on ratings are listed in Table 7.

**Table 7:** Results of repeated-measures ANOVAs, with noise level as within-subjects factor, and noise-sensitivity groups and attitude groups as between-subjects factors.

Measurement	Source	df	F	Sig.	Partial Eta Squared
E1	Within-Subjects				
	Noise level	6	147.41	.000	.88
	Noise level x Noise-sensitivity group	6	11.28	.000	.36
	Noise level x Attitude group	6	1.35	.239	.06
	Error(Noise level)	120			
	Between-Subjects				
	Intercept	1	1096.16	.000	.98
	Noise-sensitivity group	1	176.72	.000	.90
	Attitude group	1	0.06	.804	.00
	Error	20			
E2	Within-Subjects				
	Noise level	4	115.44	.000	.85
	Noise level x Noise-sensitivity group	4	13.11	.000	.40
	Noise level x Attitude group	4	1.04	.390	.05
	Error(Noise level)	73			
	Between-Subjects				
	Intercept	1	1079.61	.000	.98
	Noise-sensitivity group	1	125.84	.000	.86
	Attitude group	1	0.41	.527	.02
	Error	20			
E3	Within-Subjects				
	Noise level	6	134.87	.000	.87
	Noise level x Noise-sensitivity group	6	12.29	.000	.38
	Noise level x Attitude group	6	1.16	.332	.06
	Error(Noise level)	120			
	Between-Subjects				
	Intercept	1	969.83	.000	.98
	Noise-sensitivity group	1	130.51	.000	.87
	Attitude group	1	0.00	.991	.00
	Error	20			
E4	Within-Subjects				
	Noise level	3	133.01	.000	.87
	Noise level x Noise-sensitivity group	3	5.81	.002	.23
	Noise level x Attitude group	3	0.50	.675	.03
	Error(Noise level)	58			
	Between-Subjects				
	Intercept	1	632.73	.000	.97
	Noise-sensitivity group	1	95.95	.000	.83
	Attitude group	1	0.62	.442	.03
	Error	20			
Annoyance	Within-Subjects				
	Noise level	6	272.56	.000	.93
	Noise level x Noise-sensitivity group	6	14.94	.000	.43
	Noise level x Attitude group	6	0.61	.722	.03
	Error(Noise level)	120			
	Between-Subjects				
	Intercept	1	1261.79	.000	.98
	Noise-sensitivity group	1	64.53	.000	.76
	Attitude group	1	2.38	.139	.11
	Error	20			

**Fig 1:**
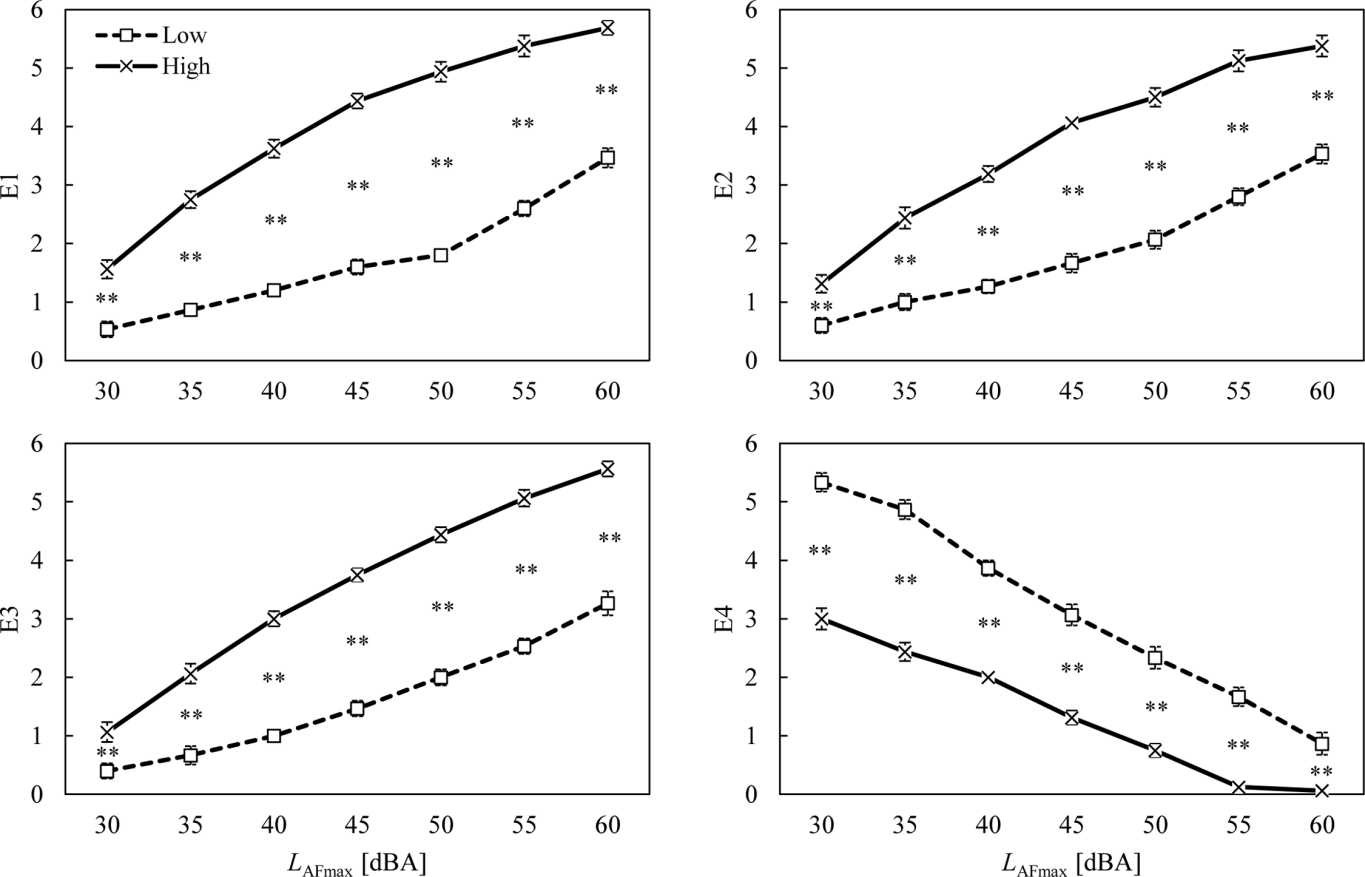
Ratings of perceived emotions for low (□) and high (×) noise-sensitivity groups as a function of noise level. Error bars indicate standard errors. Asterisks indicate significant differences between means according to the Mann-Whitney U test (*p < 0.05 and **p < 0.01).

**Fig 2:**
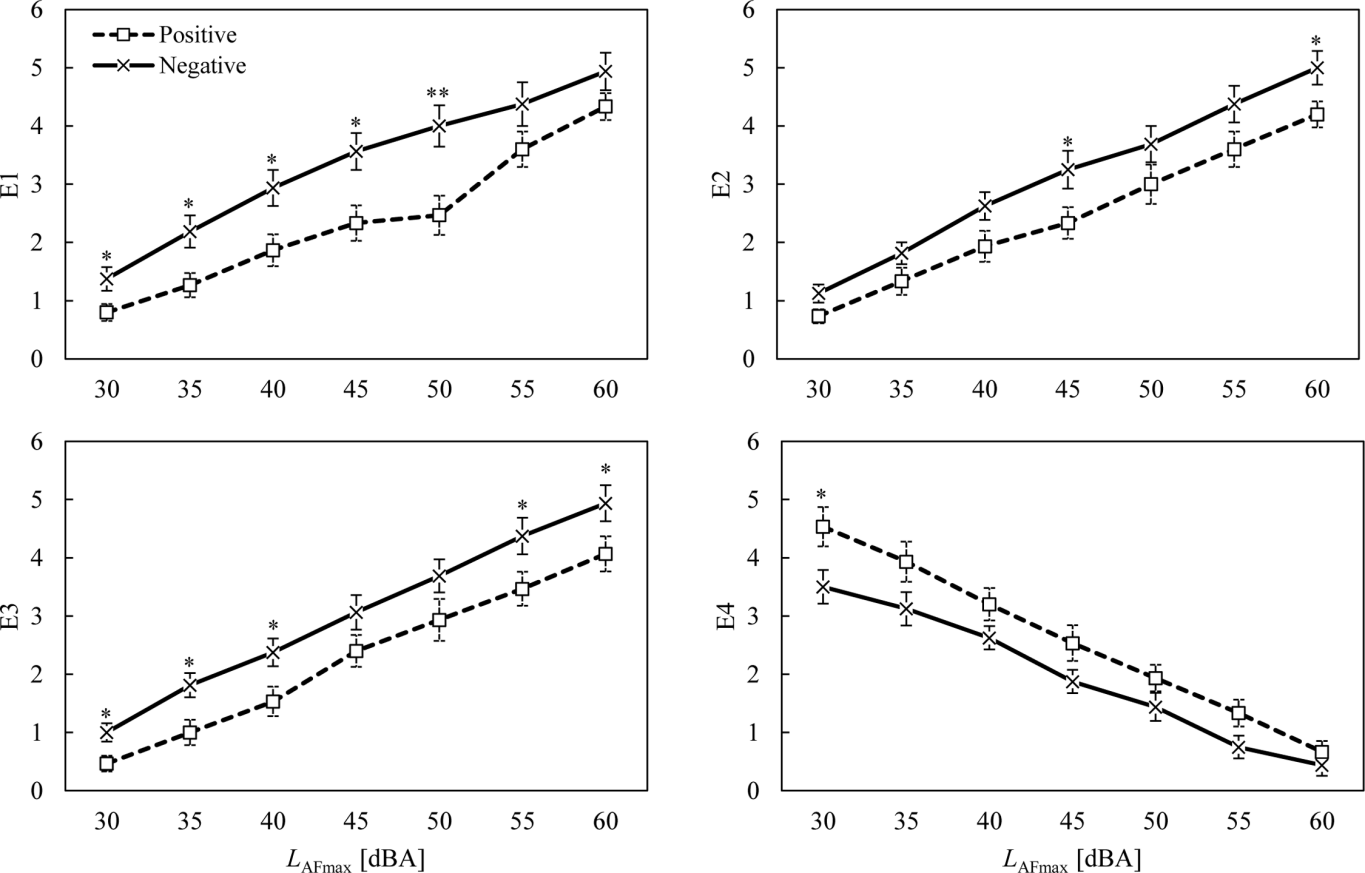
Ratings of perceived emotions for positive (□) and negative (×) attitude groups as a function of noise level. Error bars indicate standard errors. Asterisks indicate significant differences between means according to the Mann-Whitney U test (*p < 0.05 and **p < 0.01).

The high noise-sensitivity group showed greater emotion ratings for E1–E3 than the low noise-sensitivity group (Fig 1). The differences in ratings between the two groups were the smallest at the lowest noise level and they increased with an increase in noise level. Opposite tendencies were found in E4, showing that participants who were sensitive to noise tended to assign lower E4 ratings than the less sensitive participants. The results of the Mann-Whitney U tests confirmed that the emotion ratings for the high noise-sensitivity group were significantly distinct from those for the low noise-sensitivity groups, at all noise levels. Similar tendencies were found between the positive and negative attitude groups (Fig 2). Significantly different emotion ratings were found between the positive and negative attitude groups at most noise levels.

**Fig 3:**
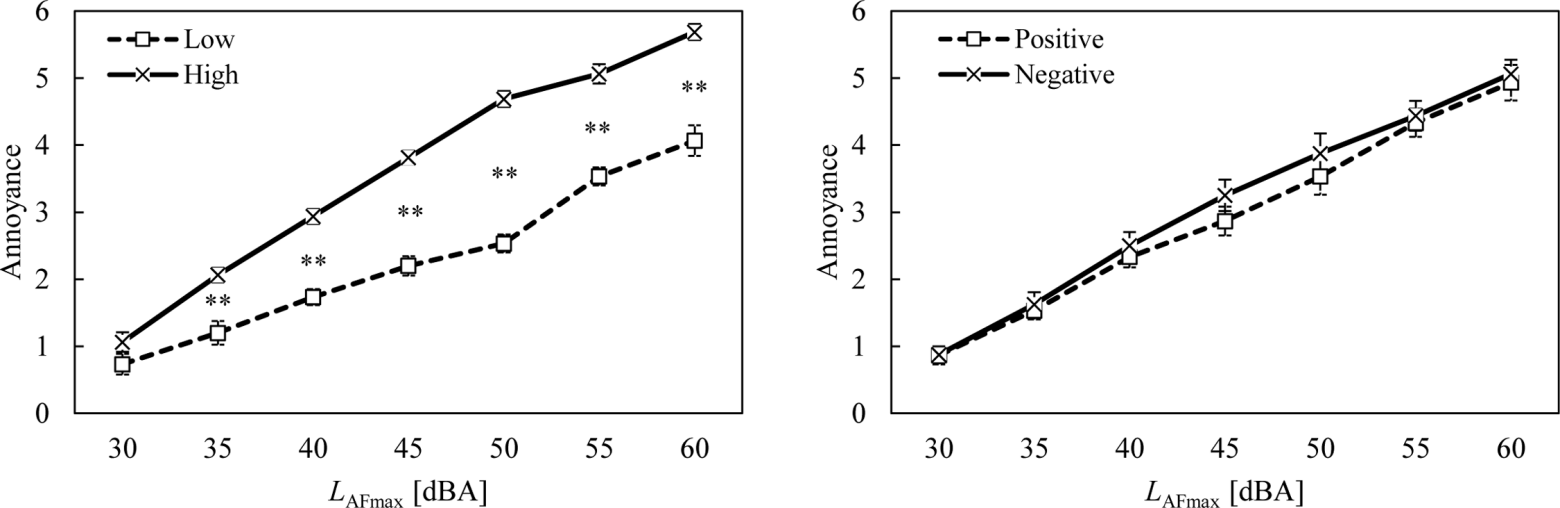
Ratings of noise annoyance for low (□) and high (×) noise-sensitivity groups and positive (□) and negative (×) attitude groups as a function of noise level. Error bars indicate standard errors. Asterisks indicate significant differences between means according to the Mann-Whitney U test (*p < 0.05 and **p < 0.01).

Fig 3 presents the noise annoyance ratings of the noise-sensitivity and attitude groups as a function of noise level. It was found that noise annoyance ratings increased with sound pressure level and that the ratings of the high noise-sensitivity group were greater than those of the low noise-sensitivity group. The high noise-sensitivity group showed steeper changes in noise annoyance than the low noise-sensitivity group. The results of the Mann-Whitney U tests indicated that the differences between the sensitivity groups were statistically significant at all levels, except for those at 30 dBA. Similar to the emotion ratings, the differences in noise annoyance ratings between the noise-sensitivity groups were more significant with louder noise. However, for the attitude groups, there was no significant difference between the positive and negative attitude groups.

**Fig 4:**
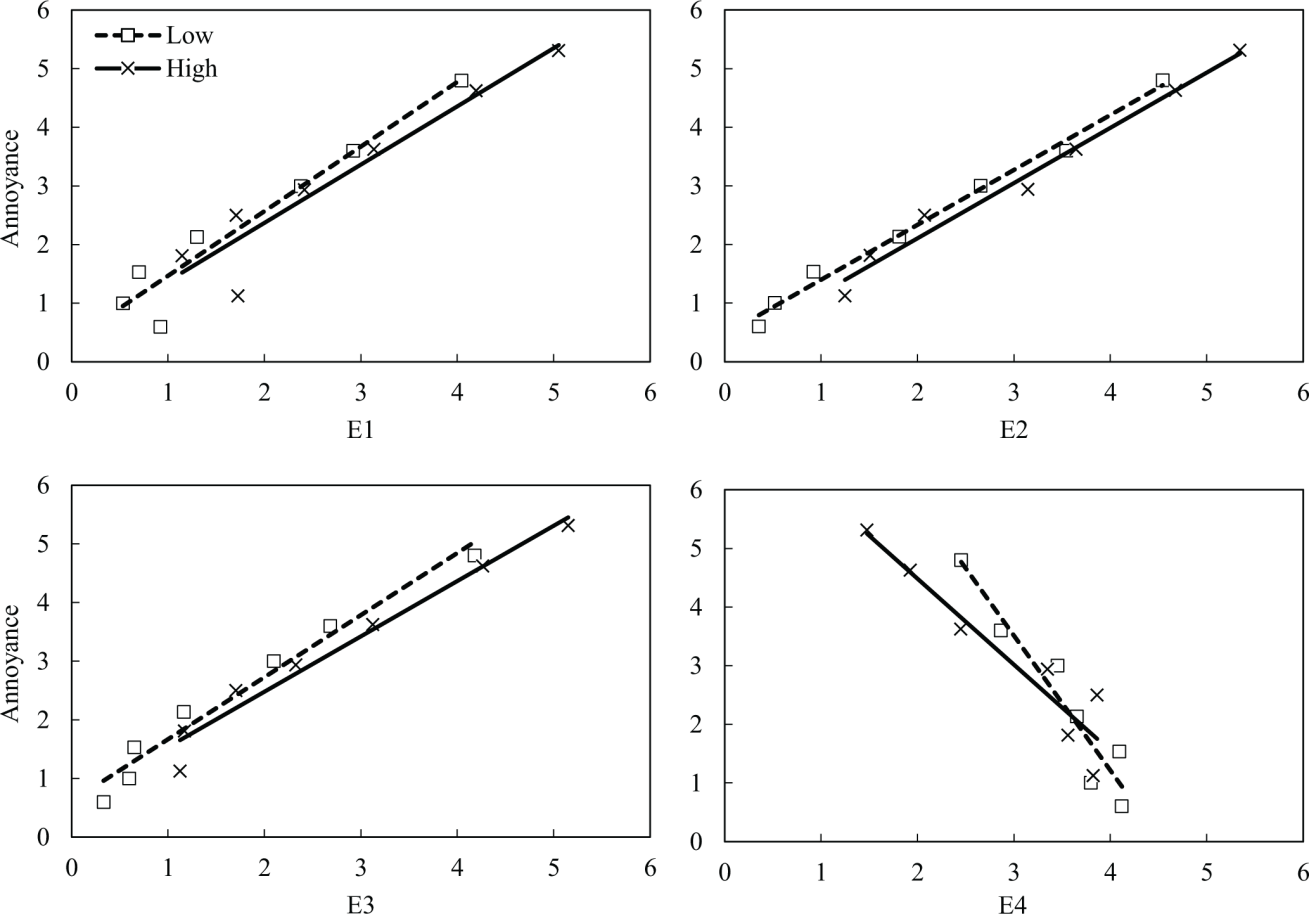
Relationships between noise annoyance and perceived emotions for low (□) and high (×) noise-sensitivity groups.

**Fig 5:**
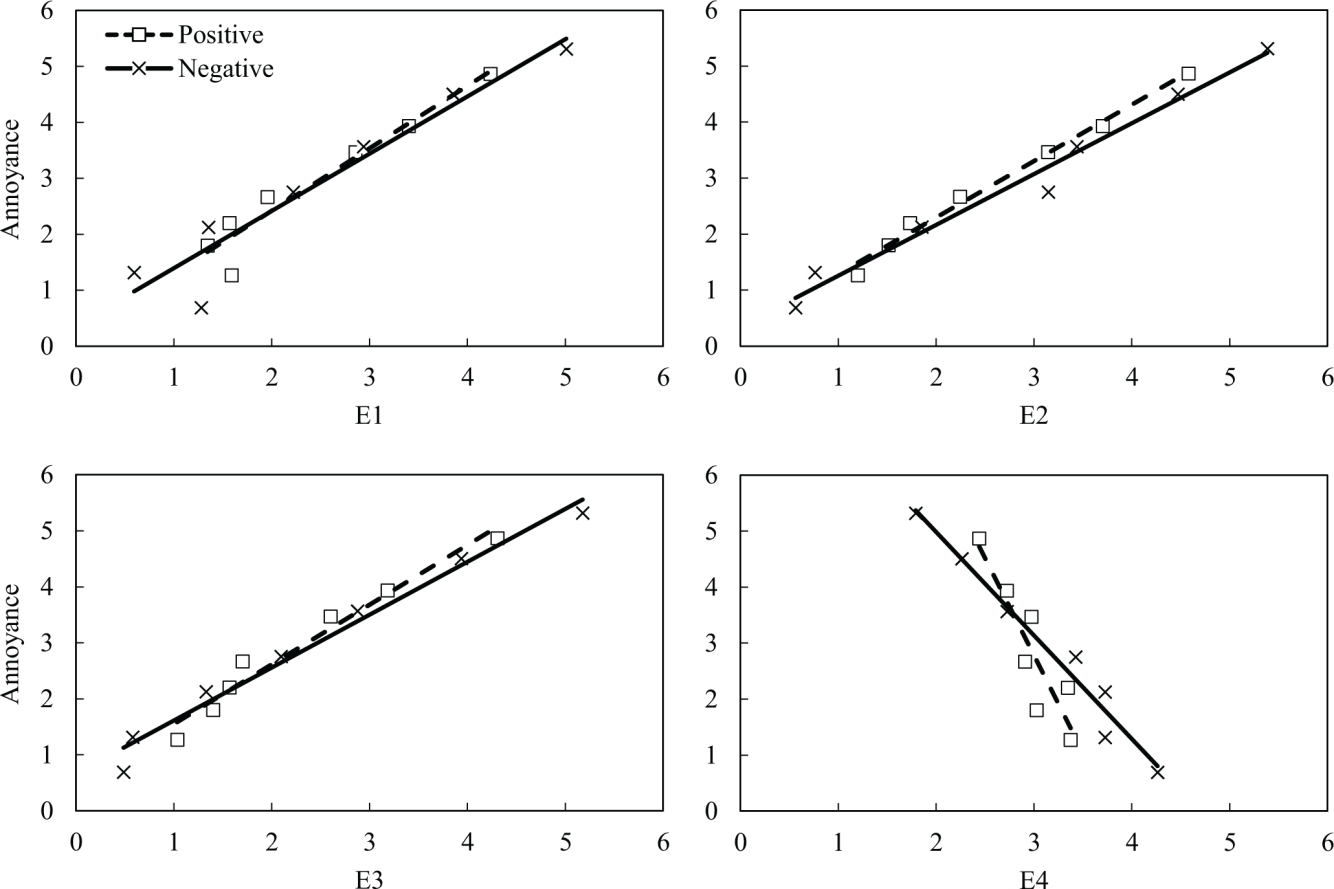
Relationships between noise annoyance and perceived emotions for positive (□) and negative (×) attitude groups.

The relationships between the ratings of emotions (E1–E4) and noise annoyance for the low and high noise-sensitivity groups are presented in Fig 4. The E1–E3 clusters containing negative emotions had positive correlations with noise annoyance, whereas the relationship between E4 and noise annoyance was negative. Fig 4 illustrates the differences between the low and high noise-sensitivity groups in terms of the range of mean ratings. For example, the ratings of E1 for the high noise-sensitivity group ranged from 1.1 to 5.1, whereas those for the low noise-sensitivity group ranged from 0.5 to 4.0. Similar patterns were observed in the relationships between the four emotion clusters (E1–E4) and noise annoyance for the positive and negative attitude groups (Fig 5). The ratings of the three clusters on negative emotions (E1–E3) were positively correlated with noise annoyance, whereas that of E4 showed negative relationship with noise annoyance. The correlation coefficients for the relationship between the ratings of emotions and noise annoyance for the positive and negative groups were similar to those for the noise-sensitivity groups.

Correlations between emotions and noise annoyance with noise level and annoyance are presented in Table 8. It also presents correlations tested with all participant responses, as well as noise sensitivity and attitude groups’ responses. It was found that all emotions and annoyance ratings showed significant correlations with noise level. It also shows that all emotions showed significant correlations with annoyance. E4 was the only variable with negative correlations and much lower coefficients than the other variables. This indicates that negative emotions have a greater association with increased noise levels, and they are more useful for understanding noise annoyance than emotion regarding empathy. Higher correlation coefficients were found from the high noise-sensitivity group and negative attitude group compared with the low noise-sensitivity group and positive attitude group. However, Fisher’s r to z transformation showed that the correlation coefficients between groups were not significantly different.

**Table 8:** Correlations of emotions (E1–E4) and noise annoyance with (a) noise level and (b) annoyance; tested between all participants, noise-sensitivity groups, and attitude groups.

(a)			E1	E2	E3	E4	Annoyance
All participants (n = 41)		Noise level	.63^**^	.70^**^	.64^**^	-.37^**^	.77^**^
Noise-sensitivity group	Low (n = 15)	Noise level	.64^**^	.74^**^	.64^**^	-.26^**^	.80^**^
	High (n = 16)	Noise level	.67^**^	.74^**^	.70^**^	-.43^**^	.77^**^
Attitude group	Positive (n = 15)	Noise level	.52^**^	.59^**^	.52^**^	-.17^*^	.70^**^
	Negative (n = 16)	Noise level	.72^**^	.80^**^	.74^**^	-.36^**^	.86^**^
(b)			E1	E2	E3	E4	Annoyance
All participants (n = 41)		Annoyance	.87^**^	.88^**^	.89^**^	-.66^**^	1
Noise-sensitivity group	Low (n = 15)	Annoyance	.87^**^	.87^**^	.81^**^	-.46^**^	1
	High (n = 16)	Annoyance	.81^**^	.85^**^	.78^**^	-.56^**^	1
Attitude group	Positive (n = 15)	Annoyance	.83^**^	.88^**^	.82^**^	-.45^**^	1
	Negative (n = 16)	Annoyance	.88^**^	.89^**^	.84^**^	-.44^**^	1

* *p* < 0.05;

** *p* < 0.01

Sample size (n) of each group was the same for all tested correlations.

**Fig 6:**
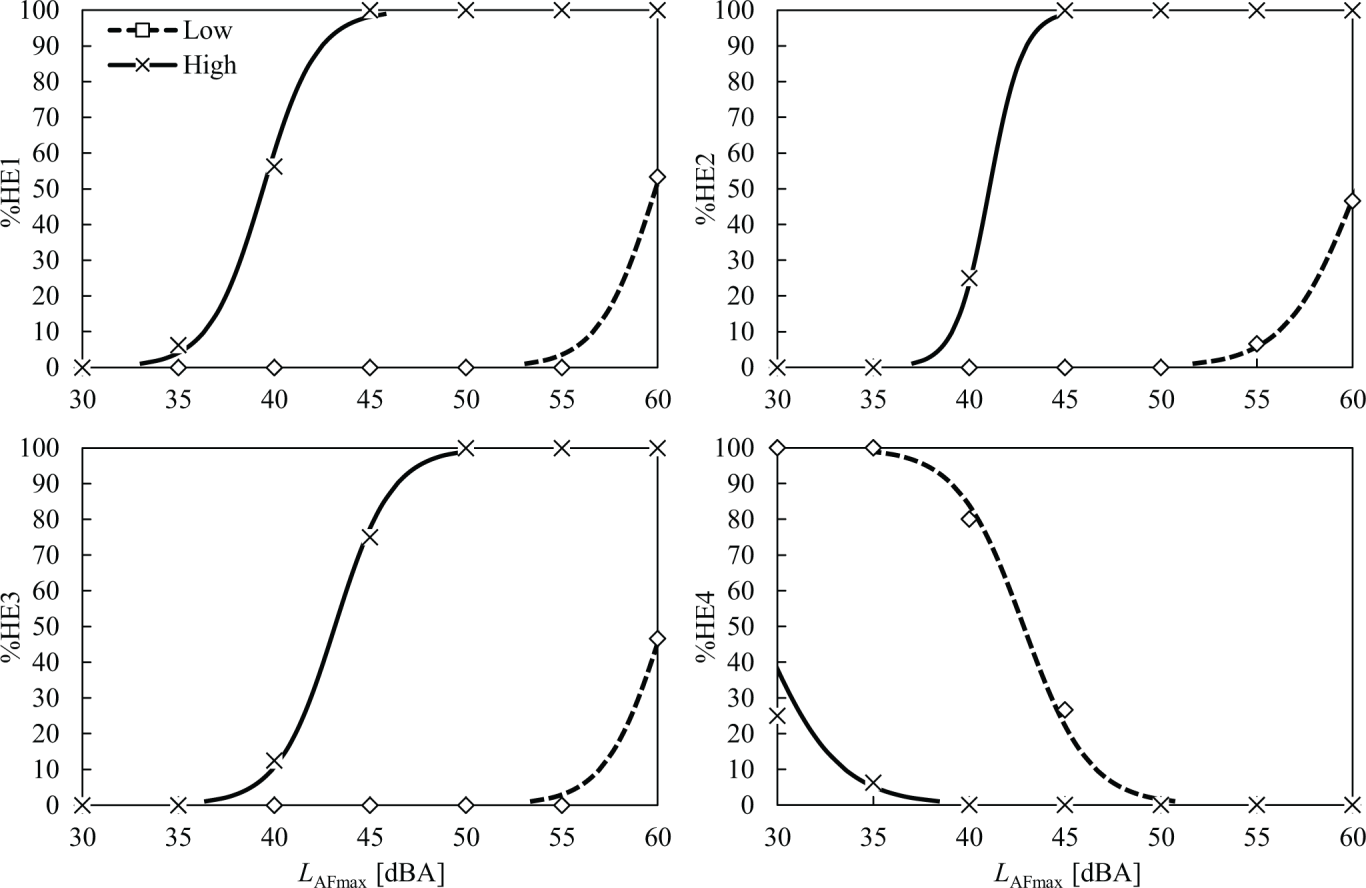
Percentage of high emotion ratings (%HE1–%HE4) for the low (□) and high (×) noise-sensitivity groups as a function of noise level. Probit regression curves are also presented for both groups.

**Fig 7:**
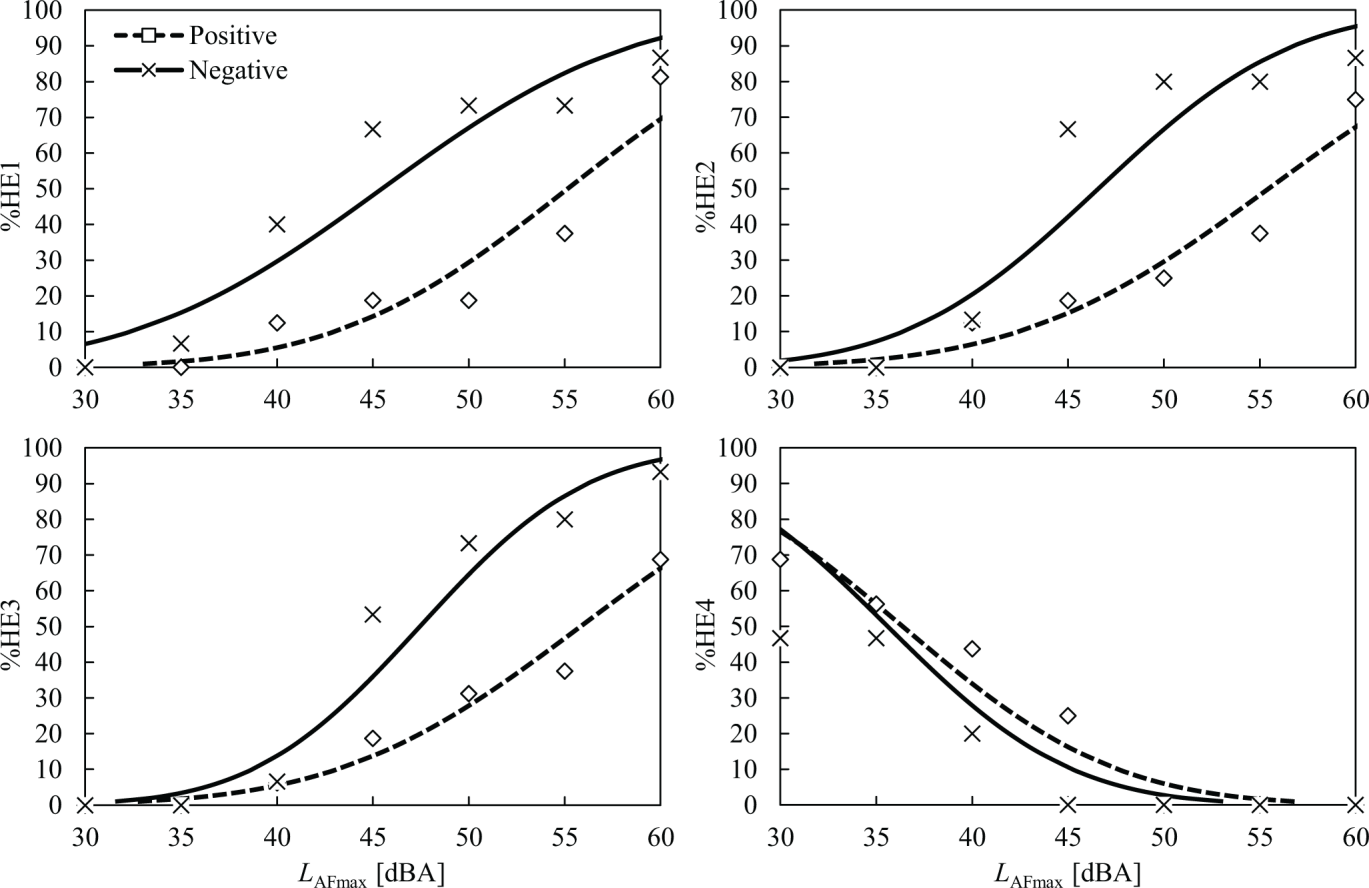
Percentage of high emotion ratings (%HE1–%HE4) for the positive (□) and negative (×) attitude groups as a function of noise level. Probit regression curves are also presented for both groups.

The percentages of high emotion ratings for the four clusters (%HE1–%HE4) as a function noise level are plotted in Figs 6 and 7. Notable differences between the noise-sensitivity and attitude groups were observed for all the emotion clusters. For the high noise-sensitivity group (Fig 6), the percentage of highly rated E1 (%HE1) started to increase above 30 dB, and it reached 100% at 45 dB. However, the low noise-sensitivity group’s %HE1 remained at 0% until 55 dB. This indicates that participants who were sensitive to noise chose rating scores above the cut-off point (5 or 6 on a 7-point numerical scale), even at low noise levels such as 35 dB. However, no one in the low noise-sensitivity group selected 5 or 6 even at 55 dB. Similar tendencies were found in the E2, E3, and E4, showing huge differences between the noise-sensitivity groups. For example, when *L*_AFmax_ was at 50 dB, the %HE2 and %HE3 were 100% for the high noise-sensitivity group, whereas they were 0% for the low noise-sensitivity group. Although the tendencies are less clear than those for the noise-sensitivity groups, the %HE1–%HE3 of the negative attitude groups were consistently higher and the %HE4 was lower than those of the positive attitude group (Fig 7).

**Fig 8:**
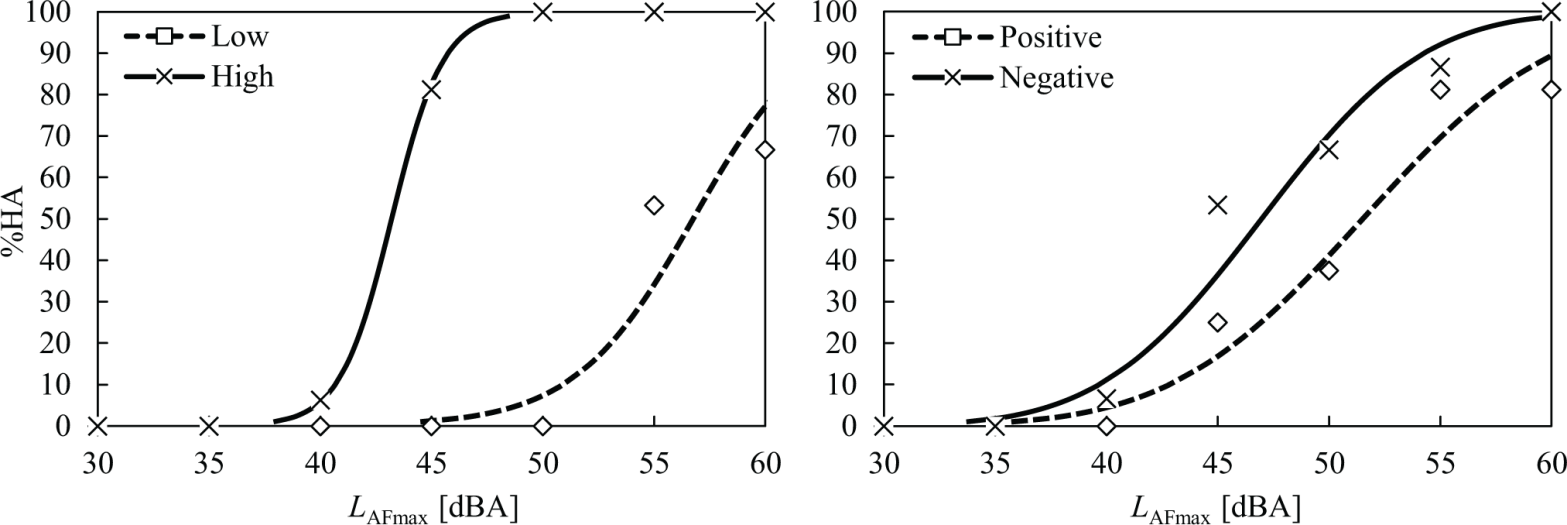
Percentage of highly annoyed (%HA) for the low (□) and high (×) noise-sensitivity groups and positive (□) and negative (×) attitude groups as a function of noise level. Probit regression curves are also presented for both groups.

Fig 8 compares the percentages for those who were highly annoyed (%HA) in the noise-sensitivity and attitude groups. For the high noise-sensitivity group, the percentage of those who were highly annoyed (%HA) increased sharply in the region between 40 dB and 45 dB, and it then reached 100% at 50 dB. In contrast, the %HAs of the low noise-sensitivity group remained at 0% until 45 dB, and it increased slowly above 50 dB. For the attitude groups, the %HA of the positive attitude group increased more slowly than that of the negative attitude group.

## Discussion

### Lexicon clusters

As described by Roseman et al. [41], emotion is caused by the way in which a person interprets a situation (i.e. appraisals). For instance, people feel pain or fear if they believe that they will not be able to resolve a negative situation satisfactorily [42]. The four emotion clusters evoked by neighbour noise may also have had a relationship with potential appraisals. The first cluster, E1, which contains the largest number of lexicons, was related to anger and hostility, mainly towards the noise source (i.e. upstairs neighbours). Anger is caused by a blocked ‘goal’ [43], which may cause the perception of the absence of a reward or presence of a punishment [44]. The term ‘goal’ is referred to as an outcome that is personally significant [45, 46]. If the noise of neighbours’ footsteps has frequently disturbed residents’ significant activities, such as sleeping and studying (i.e. ‘goal’) [32], these experiences might lead to anger-related emotions. Anger is also linked with a specific appraisal called ‘other-blame’, which is a belief that the unpleasant situation was wrongly caused by someone or something [45]. Indeed, it may be argued that residents who appraise the noise event as their upstairs neighbours’ fault or carelessness would perceive anger-related emotions towards their neighbours. The appraisal of ‘other-blame’ contains a belief that the person causing the event acted in an improper or unfair manner [14, 44]; thus, residents might perceive anger-related emotions towards their neighbours who do not act appropriately regarding the noise problem (e.g. by not apologising and continuing to make the noise). Noise-related crime among neighbours [31] could also be explained in relation to anger, which often motivates approach and attack behaviours [42].

The lexicons in the E2 cluster were related to the emotions of dislike and irritation, mostly towards the situation of noise exposure. Most lexicons in this cluster were closely related to the E1 cluster on anger. For instance, Shaver et al. [14] classified ‘irritation’ under a prototype of ‘ANGER’. However, there are two significant differences between E1 and E2. Firstly, the lexicons in E1 expressed emotions mainly towards neighbours, whereas those in E2 tended to target the situation of noise exposure. Secondly, E1 and E2 had different levels of arousal according to the structure of emotion [10, 47, 48]. Most structures of emotion (e.g. Watson and Tellegen [47] and Larsen and Diener [48]) were developed based on the suggestion made by Russell [10], who proposed the structure of emotion using a circular model comprising two dimensions (appraisal dimensions of arousal and valence). According to the dimensional models, the lexicons in E1 and E2 had different levels of arousal; lexicons in E1 showed relatively high arousal, whereas those in E2 showed lower arousal. In this study, E2 also showed greater correlations with annoyance ratings compared with the other clusters, possibly due to the semantic similarity between the lexicon ‘annoyance’ and the lexicons in E2, such as ‘bothered’ or ‘irritated’. Guski et al. [49] examined the concept of noise annoyance in different languages and listed ten expressions which were rated similarly to ‘annoyance’. Most of them overlapped with the lexicons in E2, such as ‘get on my nerves’, ‘irritated’, and ‘vexed’.

The third cluster (E3) mainly contained lexicons representing physical and emotional pains. Shaver et al. [14] explored various lexicons expressing general emotions and grouped them into six groups including ‘ANGER’ and ‘SADNESS’. In particular, ‘SADNESS’ included a subgroup expressing pain, such as ‘suffering’ and ‘hurt’. However, in this study exploring the emotions evoked by a particular type of noise, pain was found to be one of the main emotion clusters. The emotion lexicons expressing sadness were initially included in the 120 collected lexicons, but they were not chosen often by the respondents in Survey I; consequently, they were not included in the 60 lexicons used in Survey II. This implies that the exposure to neighbours’ footstep noise may not elicit the emotion of sadness to a considerable extent. In contrast to the emotions in E1 and E2 targeting the noise source and situation of noise exposure, respectively, the lexicons in E3 expressed the physical and emotional pain perceived by the respondents. For example, the lexicon ‘vengeful’ (E1) was directed towards the upstairs neighbours who were responsible for making the noise, and the lexicon ‘unpleasant’ (E2) was directed towards the situation that the respondent was exposed to the noise. On the other hand, ‘feeling sick’ (E3) was an expression that described what the respondent felt or perceive inwardly. These findings are in line with the results of a previous study suggesting that neighbour noise evokes outwardly directed aggression and inward reactions such as tension and feelings of pressure [17]. Given that many lexicons in E3 described physical pain (e.g. my head is throbbing, feeling sick, tired), this finding added further evidence to a previous finding that floor impact noise increases health complaints [25, 32].

The fourth cluster (E4) contained expressions narrated by residents who understood the situation of the noise event or their neighbours’ circumstances of making noise, or by those who did not care much about the noise exposure. The lexicons in E4 expressed sympathy and empathy. According to Wispé [50], sympathy is a way of relating to others, which refers to an increased awareness of another person’s plight as something to be alleviated, and therefore, it leads to an unselfish concern for the person. On the other hand, empathy is a way of knowing, which is an attempt of understanding the subjective experiences of another person without prejudice [50]. Some lexicons in E4 could be used to express sympathy. However, the other lexicons implied indifference or mere knowing and understanding, which comprise empathy [51, 52] rather than sympathy. Thus, the prototype E4 was labelled as ‘EMPATHY’. In addition, empathy has been suggested to weaken annoyance as well as a vigilance coping strategy (e.g. making noise complaints) [32]. Thus, respondents who tended to exhibit higher ratings on the empathy-related cluster might have been likely to indicate having a lower level of annoyance. Moreover, it is unlikely for them to choose a vigilance coping strategy, as it may lead to conflicts with their neighbours [32]. This is in agreement with the findings of Zaki and Ochsner [53] who suggested that individuals often want to approach empathy when it facilitates important social goals, such as relationship formation and maintenance. Here, the relationship between neighbours can also be considered a variable that has a strong influence on empathy. Park et al. [32] proposed that the relationship with one’s neighbours is an intervening condition that influences one’s perception of noise events. It can be assumed that residents who have close and positive relationships with their neighbours may say ‘there is no reason to get angry’ (E4) regarding a noise event. In such situations, empathy develops through the positive relationship with one’s neighbours. However, intervening conditions may decrease people’s empathy when it is painful or costly, or when they interact with outgroup targets [53].

### Intervening conditions

As mentioned earlier, intervening conditions cannot be overlooked when it comes to explaining emotions. Appraisals influence the emotions that are evoked [41], while other variables influence the procedure of appraisal. For example, residents who have a negative relationship with their neighbours may perceive less empathy and more negative emotions. The present study tested two intervening conditions which were suggested to have reciprocal relationships with the perception of and reaction to floor impact noise [32]: noise sensitivity and attitude towards neighbours. Guski [54] also emphasised that noise sensitivity, personal evaluations of the source, coping capacity, general attitude, history of noise exposure, and residents’ expectations have important influences on noise annoyance. This study confirmed the findings of previous studies [24, 28, 32, 54], showing notably different trends between noise-sensitivity groups and attitude groups. Ryu and Jeon [24] highlighted a significant impact of noise sensitivity on the annoyance evoked by indoor residential noises. Park et al. [28] also found significant differences in the annoyance ratings for floor impact noise between low and high noise-sensitive participants via a laboratory experiment. Similarly, the present study found significant differences between the noise-sensitivity groups, which imply that higher noise sensitivity would influence individuals’ appraisals to perceive higher anger, dislike, and pain, whereas low noise sensitivity may lead to a more empathetic appraisal of the event. This study also revealed that attitudes towards neighbours had an influential role in emotion and annoyance-evoking appraisals. Although there are clearer differences between the noise-sensitivity groups than the attitude groups presented in Figs 1 and 2, one cannot conclude that attitude did not have a significant role in emotional responses. Since Figs 1 and 2 illustrate the emotional changes in groups as the noise levels increased, it is reasonable to conclude that noise sensitivity was a grouping factor that reveals clear differences between the groups. Contrary to expectations, the attitude towards noise did not have a significant impact on emotions and noise annoyance. However, this could be further investigated by using more appropriate questions covering all different attitudes. The present study found that the participants with positive attitudes towards their upstairs neighbours provided lower negative emotion ratings (E1–E3), lower annoyance ratings, and higher empathy (E4). This result is consistent with the findings of Pedersen and Persson Waye [55], who revealed that the annoyance induced by wind turbine noise was affected by negative attitudes towards wind turbines.

### Annoyance and emotions

Several studies have conducted questionnaire surveys and laboratory experiments to evaluate noise annoyance because it has been a most popular measure of the adverse effect of noise on individuals and on the community [21, 49, 54]. However, for neighbour noise, which frequently causes neighbour disputes including violence [30–32], it is appropriate to measure emotions evoked by neighbour noise because annoyance cannot adequately explain or predict potential disputes or relational problems between neighbours. Therefore, the measurement of emotions using lexicons targeting the noise source (i.e. E1) or the situation of noise exposure itself (i.e. E2) would be useful to determine the perceptual dimension of the respondents and to predict their future coping strategies. In practice, some of the clusters can be selected for the prompt measurement of emotional responses to noise. In particular, it would be quite useful to measure emotions using the E1 (‘ANGER’) and E4 (‘EMPATHY’) clusters rather than the E2 (‘DISLIKE’) and E3 (‘PAIN’) clusters, for the following reasons. First, given that emotion measurements aim to predict respondents’ internal perceptions and future coping strategies, anger, an emotion which may develop into violent behaviours [42], needs to be measured, particularly in the case of noise issues between neighbours. Second, empathy needs to be assessed to predict future coping strategies. Empathy leads individuals to build or maintain positive relationships with others [53]. In particular, the present study found that empathy had the weakest correlation with annoyance. Since the measurement of annoyance cannot predict empathy, it needs to be assessed to yield extended insight into respondents’ perception and to predict their future coping strategies. Third, E2 (‘DISLIKE’) can be excluded because it is strongly correlated with annoyance and because most of the lexicons in E2 were similar to annoyance [49]. Fourth, the lexicons in E3 (‘PAIN’) described pain, but as discussed earlier, some of them referred to health complaints [25, 32] and they may actually contain or be connected to some other emotions (e.g. anger and fear). Finally, correlations between E1, E2, and E3 and annoyance were consistently high (Table 8). However, Fisher’s r to z transformation revealed that all the correlation coefficients were not statistically different [56]. This indicates the three emotion clusters ultimately measured the same construct (i.e. negative emotion), implying that the three clusters are interchangeable or that one cluster can cover most of what the other clusters would measure [57].

### General discussion

Previously Namba et al. [18] used lexicons in an experiment to measure the impressions of sound stimuli. In particular, they collected a pool of Japanese adjectives from a preliminary experiment, but details about the experimental procedure (e.g. level of the stimuli) and main findings (e.g. the number of adjectives) were not explained. In contrast, the present study was designed carefully to collect appropriate lexicons. In this study, the lexicons were collected from two sources: (1) interview transcripts and (2) posts in online communities. The interview transcripts were chosen as a source because the interviews were conducted using grounded theory [32]. Based on the grounded theory methodology, the interviews were conducted until the researcher was confident that no more new findings could be obtained, so that theoretical saturation was attained [58]. Therefore, it was assumed that the interview transcripts included all the possible aspects of footstep noise and residents’ reaction to the noise. The present study used a number of online posts from 18 online communities as another source of lexicons. From a number of online posts, only lexicons expressing emotions evoked by footstep noise were filtered and collected. Therefore, it can be said that the collected lexicons represent the narratives of residents adequately, particularly regarding footstep noise.

During the two online surveys, the respondents were asked to listen carefully to the noises via headphones. It was not possible to control the quality of sound reproduction, noise level, and background noise level. In particular, most headphones are limited to reproducing low-frequencies below 50 Hz. Thus, these practical constraints might have influenced the participants’ responses and the selection of the lexicons. However, all participants had experienced footstep noise from their upstairs neighbours. Therefore, they were expected to rate the lexicons based on their previous experiences and their experiences with hearing the noises via headphones. Nonetheless, the sound reproduction system (loudspeaker and subwoofer) used in the laboratory experiment is considered to be more useful to evoke people’s emotions related to footstep noise, including low-frequency components. Therefore, in the future, a small-scale study could be conducted to validate the selection of lexicons in the laboratory.

Based on the present findings, the following recommendations for future research are made. First, it would be useful to examine the emotions evoked by different types of noises. For instance, neighbour noise comprises several other noise sources. Emotions evoked by other types of floor impact noise (e.g. chair/furniture scraping) or airborne noises (e.g. voice/conversations of neighbours) can also be examined to identify any influences of different noise sources. It can also be assumed that different floor materials, shoe types, and body sizes would induce different footstep noises [59]; thus, different footstep noise stimuli may also evoke different emotional responses as well. Moreover, the concept of total annoyance can be utilised in future neighbour noise studies. It is known that the annoyance response to a single noise source and the total annoyance evoked by combined noises differ [60, 61]. Similarly, it is expected that the annoyance and emotional responses evoked by a general term of floor impact noise or neighbour noise would be different from those evoked by different single sources (e.g. footsteps, dropping of items). For example, it can be assumed that a general floor impact noise (i.e. combined) would elicit higher anger and annoyance than exposure to footstep noise alone. Second, there are several emotion lexicons in different languages and they contain different nuances of emotions. Moreover, cultural differences in emotion will yield further insights [62, 63]. Given that neighbour noise is not a problem in Korea alone, emotion research using lexicons in different languages and cultures would yield further insight into emotional responses to this type of noise. Third, different attempts could be made to evoke emotions. Participants are highly likely to become passive observers if they receive one-way, simplified, and well-controlled stimuli [64], as human emotions naturally occur in interaction with others and external events [65]. As this study presented stimuli and did not ask participants to engage or interact with anything, the participants might have responded to the questionnaire as passive observers. Thus, it is difficult to define to which extent emotions resulted from stimuli, and this study may have missed some important emotional processing factors [64]. Further consideration of methods for evoking emotional responses to neighbour’s noise in a more engaging and ecological way could be examined in the future [66]. Fourth, different methods could be used to measure emotions. Emotion lexicons are linguistic expressions, so there is a possibility that they could be either understated or overstated by the respondents. Moreover, emotion lexicons may not fully reflect the perceivers’ true emotional status, especially if they are not aware of their real inward feelings. Thus, a questionnaire survey can be provided along with measurements of physiological responses because brain and bodily functions are strongly synchronised by emotion-evoking stimuli [64]. In addition, assessments in performance settings may be of use to understand the subjective states of participants affected by noise through pre/post-test assessments. Questionnaire scales have been commonly used to assess subjective responses, but this type of scale primarily measures conscious feeling states, which only represent a partial expression of some underlying emotional process [67]. One important advantage of a performance setting is that demanding tasks elicit various stress responses, such as anxiety and worry, which facilitates an examination of subjective states [67]. Therefore, future research could be carried out in performance settings and could assess subjective states before and after eliciting emotional experiences through noise.

## Conclusion

In the present study, lexicons expressing emotions induced by neighbours’ footstep noise were collected and emotional responses were assessed in a large sample of participants hearing noises. Throughout the study, noise stimuli were used to simulate neighbours’ footstep noise particularly that made by a child and an adult. First, a total of 120 Korean lexicons were chosen from interview transcripts and online community posts. The number of lexicons was reduced to 60 through an online survey. Participants in the first survey were residents of multi-family housing buildings (*n* = 133) and they were asked to choose appropriate lexicons expressing their emotions while listening to the noises. Subsequently, another online survey was conducted with 89 residents, who rated the appropriateness of each lexicon while hearing the noises. Based on their responses, the lexicons were classified into four clusters. Negative emotions related to anger, dislike, and pain were grouped in three different clusters (E1–E3), while lexicons expressing empathy were grouped in E4. In the laboratory experiment, twenty lexicons were presented to the participants (*n* = 41), and they were asked to rate each lexicon based on their feelings during noise exposure. Noise stimuli were presented at noise levels between 30 and 60 dBA (*L*_AFmax_), in 5 dB intervals. It was found that the emotion and noise annoyance were significantly affected by noise levels, indicating that greater noise levels led to greater negative emotions and annoyance ratings. Because exposure to noise causes negative reactions such as annoyance, the three clusters representing negative emotions were strongly correlated with noise annoyance. The emotion cluster expressing ‘DISLIKE’ (E2) showed the strongest correlation with noise annoyance, whereas that expressing ‘EMPATHY’ (E4) showed the weakest correlation with noise annoyance. This study also tested whether noise sensitivity and attitudes were moderators influencing emotional responses and annoyance ratings. Both noise sensitivity and attitudes were found to significantly affect emotional responses. In particular, it was revealed that there were clear gaps between the low and high noise-sensitivity groups’ level of emotions and annoyance. Overall, the present findings suggest that lower noise level, lower noise sensitivity, and more positive attitudes towards neighbours would evoke less negative emotions and annoyance when neighbours’ footstep noises are heard. This study provides evidence that can be used in dealing with neighbour disputes and in preventing such problems in advance. Noise levels can be reduced by helping residents become aware of which activities make loud noises (e.g. children’s jumping and running [68]) and when people tend to perceive these noise events as louder. For instance, people tend to complain more about noise exposure at night or early in the morning when ambient noise levels are relatively low [32]. The findings of this study could also be used by the management office, mediation services, and local authorities. Once they address the dispute, the residents’ emotions evoked by floor impact noise could be assessed with noise sensitivity and attitude measurements with respect to the noise source. They can then interpret the severity of the negative perception and could estimate noise exposure levels based on the relationship between the noise level and emotions. An understanding of the emotional status of residents and its relation to noise levels could be useful for solving disputes between neighbours.

## Supporting information

S1 Table Keywords used for searching online postings. (DOCX)

S1 File Questions used in the laboratory experiment. (DOCX)

S2 File Responses collected from the laboratory experiment. (XLSX)
