# Supplementary material for: Emotions evoked by exposure to footstep noise in residential buildings
Source: PLoS One. 2018 Aug 13;13(8):e0202058. doi: 10.1371/journal.pone.0202058 (PMC6089415; doi:10.1371/journal.pone.0202058)
Supplement: S1 Table — (DOCX) [file pone.0202058.s001.docx]

S1 Table. Keywords used for searching online postings.

| Category | Keywords (English) | Keywords (Korean) [pronunciation] |
| --- | --- | --- |
| Main keyword | noise | 소음 [so-eum] |
|  | sound | 소리 [so-ri] |
| Sub-keyword | floor | 바닥 [ba-dak] |
|  | between floors, inter-floor | 층간 [cheung-gan] |
|  | neighbour | 이웃 [i-ut] |
|  | upstairs | 윗집 [wit-jib] |
|  | foot, footsteps | 발 [bal] |
|  | running, jumping | 뛰는 [ttwi-neun] |
|  | walking | 걷는 [geod-neun] |

Supplemental material: Table 2. Twenty lexicons used in the laboratory study.

| Emotion cluster | Lexicon (English) | Lexicon (Korean) |
| --- | --- | --- |
| E1 | unhappy | 불만스럽다 |
|  | detestable | 괘씸하다 |
|  | can’t understand | 이해가 안된다 |
|  | get enraged | 열 받는다 |
|  | ridiculous | 기가 막힌다 |
| E2 | bothered | 신경쓰인다 |
|  | unwelcome | 달갑지 않다 |
|  | dislike | 싫다 |
|  | get on my nerves | 예민해진다 |
|  | awkward | 거북하다 |
|  | vexed | 신경질난다 |
| E3 | suffering | 괴롭다 |
|  | tired | 피곤하다 |
|  | my head is throbbing | 머리가 지끈거린다 |
|  | painful | 고통스럽다 |
| E4 | bearable | 견딜 만하다 |
|  | just being patient | 그냥 참는다 |
|  | tolerable | 참을 만하다 |
|  | no reason for discomfort | 불편한 정도는 아니다 |
|  | think of it as usual | 그러려니 한다 |
