# Supplementary material for: Emotions evoked by exposure to footstep noise in residential buildings
Source: PLoS One. 2018 Aug 13;13(8):e0202058. doi: 10.1371/journal.pone.0202058 (PMC6089415; doi:10.1371/journal.pone.0202058)
Supplement: S1 File — (DOCX) [file pone.0202058.s002.docx]

| **Questions used in the laboratory experiment** |
| --- |
|  |
| Age |
|  |
|  |
| Gender |
| Male |
| Female |
|  |
| Child(ren) below 12 years old living with you |
| Yes |
| No |
|  |
| Noise sensitivity: 21 questions (Weinstein, 1978) |
| *All below questions used 6-point scales: (Strongly agree) 1 – 2 – 3 – 4 – 5 – 6 (Strongly disagree)* |
| How much do you agree with the following statements? |
| 1. I wouldn't mind living on a noisy street if the apartment I had was nice. |
| 1. I am more aware of noise than I used to be. |
| 1. No one should mind much if someone turns up his stereo full blast once in a while. |
| 1. At movies, whispering and crinkling candy wrappers disturb me. |
| 1. I am easily awakened by noise. |
| 1. If it's noisy where I'm studying, I try to close the door or window or move someplace else. |
| 1. I get annoyed when my neighbours are noisy. |
| 1. I get used to most noises without much difficulty. |
| 1. It matters to me if an apartment I were interested in renting was located across from a fire station. |
| 1. Sometimes noises get on my nerves and get me irritated. |
| 1. Even music I normally like will bother me if I'm trying to concentrate. |
| 1. It wouldn't bother me to hear the sounds of everyday living from neighbours (footsteps, running water, etc. |
| 1. When I want to be alone, it disturbs me to hear outside noises. |
| 1. I'm good at concentrating no matter what is going on around me. |
| 1. In a library, I don't mind if people carry on a conversation if they do it quietly. |
| 1. There are often times when I want complete silence. |
| 1. Motorcycles ought to be required to have bigger mufflers. |
| 1. I find it hard to relax in a place that's noisy. |
| 1. I get mad at people who make noise that keeps me from falling asleep or getting work done. |
| 1. I wouldn't mind living in an apartment with thin walls. |
| 1. I am sensitive to noise. |
|  |
| Attitude towards their upstairs neighbours: 6 questions (Park et al., 2018) |
| *All below questions used 6-point scales: (Strongly agree) 1 – 2 – 3 – 4 – 5 – 6 (Strongly disagree)* |
| How do you feel about your upstairs neighbours? |
| 1) They are good people. |
| 2) I am happy to be their neighbour. |
| 3) We understand each other in many things. |
| 4) I know and understand their situation very well. |
| 5) We greet each other with a friendly hello. |
| 6) They try not to make as much noise as possible for us. |

| Self-rated emotion (20 questions) and annoyance (1 question) |
| --- |
| *All below questions used 7-point scales: (Not at all) 0 – 1 – 2 – 3 – 4 – 5 – 6 (Extremely)* |
| Please rate the extent to which each lexicon is appropriate for expressing your emotions toward the noise you are currently hearing. |
| 1. unhappy |
| 1. detestable |
| 1. can’t understand |
| 1. get enraged |
| 1. ridiculous |
| 1. bothered |
| 1. unwelcome |
| 1. dislike |
| 1. get on my nerves |
| 1. awkward |
| 1. vexed |
| 1. suffering |
| 1. tired |
| 1. my head is throbbing |
| 1. painful |
| 1. bearable |
| 1. just being patient |
| 1. tolerable |
| 1. no reason for discomfort |
| 1. think of it as usual |
| 1. annoying |

Park, S. H., Lee, P. J., & Jeong, J. H. (2018). Effects of noise sensitivity on psychophysiological responses to building noise (under review). Building and Environment.

Weinstein, N. D. (1978). Individual differences in reactions to noise: a longitudinal study in a college dormitory. Journal of Applied Psychology, 63(4), 458-466.
